# Integrative taxonomy methods reveal high mealybug (Hemiptera: Pseudococcidae) diversity in southern Brazilian fruit crops

**DOI:** 10.1038/s41598-017-15983-5

**Published:** 2017-11-16

**Authors:** Vitor C. Pacheco da Silva, Mehmet Bora Kaydan, Thibaut Malausa, Jean-François Germain, Ferran Palero, Marcos Botton

**Affiliations:** 1 Plant Protection Graduate Program, Plant Protection Department, UFPel, Pelotas, RS Brazil; 2 Imamoglu Vocational School, Çukurova University, Adana, Turkey; 3 Çukurova University, Biotechnology Research and Application Centre, Adana, Turkey; 4 INRA, Univ. Nice Sophia Antipolis, CNRS, UMR 1355–7254 Institut Sophia Agrobiotech, 06900 Sophia, Antipolis France; 5 Anses, Laboratoire de la Santé des Végétaux, Unité d’Entomologie et Plantes Invasives, Montferrier-sur-Lez, France; 6 Dept. Marine Ecology, Centro de Estudios Avanzados de Blanes, Blanes, Spain; 7 Embrapa Uva e Vinho, Bento Gonçalves, RS Brazil

## Abstract

The Serra Gaúcha region is the most important temperate fruit-producing area in southern Brazil. Despite mealybugs (Hemiptera: Pseudococcidae) infesting several host plants in the region, there is a lack of information about the composition of species damaging different crops. A survey of mealybug species associated with commercial fruit crops (apple, persimmon, strawberry and grapes) was performed in Serra Gaúcha between 2013 and 2015, using both morphology and DNA analyses for species identification. The most abundant species were *Pseudococcus viburni* (Signoret), found on all four host plant species, and *Dysmicoccus brevipes* (Cockerell), infesting persimmon, vines and weeds. The highest diversity of mealybug species was found on persimmon trees, hosting 20 different taxa, of which *Anisococcus granarae* Pacheco da Silva & Kaydan, *D*. *brevipes*, *Pseudococcus sociabilis* Hambleton and *Ps*. *viburni* were the most abundant. A total of nine species were recorded in vineyards. *Planococcus ficus* (Signoret) and *Pseudococcus longispinus* (Targioni Tozzetti) were observed causing damage to grapes for the first time. A single species, *Ps*. *viburni*, was found associated with apples, while both *Ps*. *viburni* and *Ferrisia meridionalis* Williams were found on strawberry. Four of the mealybug species found represent new records for Brazil.

## Introduction

Brazil is the third largest fruit producer in the world, with a cultivated area of 2.5 million hectares and an estimated production of 40 million tons^1,2^. Fruit crops have a high social importance in different regions of the country (e.g. nearly six million employees involved^2^), particularly in the Southern Region, which is responsible for the largest temperate fruit production^3^. Within this area, the Serra Gaúcha Region of the Rio Grande do Sul State (RS) includes vineyards (*Vitis* spp.) for wine and juice production, and apple orchards (*Malus domestica* Borkh) as the most important crops^3^, but with other crops being of local importance. For example, vineyards in Rio Grande do Sul cover about 50,000 ha, apple trees about 17,493 ha and persimmon (*Diospyros kaki* L.) around 2,239 ha^4^.

The presence of agricultural pests is one of the main factors limiting fruit production in Brazil. The value of yield losses caused by insects is around 1.6 billion dollars annually^5^. Chemical insecticides, applied mainly to control fruit flies and moths, still represent the primary form of control in the Serra Gaúcha Region, although recent progress has been achieved by alternative pest-control technologies, such as the use of pheromones, toxic baits and mass trapping^6–8^. Chemical control through insecticides is often ineffective and it may have an indirect effect on non-target pests, mainly due to its negative impact on natural enemy populations.

Scale insects (Hemiptera: Coccomorpha) are important agricultural pests that can develop on fruits, leaves, branches, trunk and roots, infesting a range of host plants such as vineyards and fruit orchards^9,10^. The diversity of scale insect species is high, with 148 species recorded on grapevines, 69 on persimmon trees, 28 on strawberry (*Fragaria x ananassa* Duchesne) and 26 on apple trees^11^. Most adult females and nymphs extract large amounts of phloem sap while excreting the excess of water and sugar as honeydew^12,13^. This sugary substance then falls on leaves and fruits, serving as a substrate for the development of sooty mold fungi. Furthermore, cosmetic damage is caused to fruits as a result of scale insect infestation (e.g. by mealybugs (Pseudococcidae)), depreciating its value or even rendering it unmarketable. Mealybugs may hamper international trade to several markets due to quarantine restrictions^10^. Additionally, mealybugs are responsible for virus transmission of diseases, such as grapevine leafroll-associated viruses (GLRaVs)^10,14^. Scale insects have been associated with damaged fruit previously in Brazil^15,16^. In southern Brazil, mealybug population outbreaks are becoming increasingly common^17^, and preliminary reports have been carried out on vineyards^16,18^, but the lack of information on mealybugs currently threatens any prospects for integrated pest management in the area.

Correct identification of mealybug species is of utmost importance for the establishment of adequate pest management programs, especially when aiming to implement specific control techniques, such as the use of sex pheromones or biological control. Mealybug identification is usually carried out by microscopic analysis of morphological structures present on the body surface of the adult female. However, the presence of cryptic speciation and significant intraspecific morphological variation makes identification particularly challenging, especially for non-specialists^19^. In this case, DNA analysis can be an auxiliary tool to facilitate insect identification. The “DNA barcoding” international project proposed by Hebert *et al*.^20^ is based on the molecular characterization of one fragment of the Cytochrome oxidase subunit I (COI) mitochondrial gene, and has been used successfully for many animal taxa. However, due to the high intraspecific variation observed in the COI gene for mealybugs, the use of alternative gene regions has been favored^21,22^. In particular, the 28S ribosomal DNA region has proved useful and complementary to COI due to its ability to discriminate between species while maintaining low intraspecific variation^23^. In the present study, both morphological and DNA analysis (using COI and 28S gene regions) were used to characterize mealybug species infesting fruit orchards in the Serra Gaúcha region. Different crops, including apples and persimmon orchards, strawberry fields and vineyards were surveyed, including the weeds in the same cropped area. The comprehensive sampling enabled us to identify specific associations between mealybugs and their host plants.

## Results

### Morphological identification

**Figure 1:**
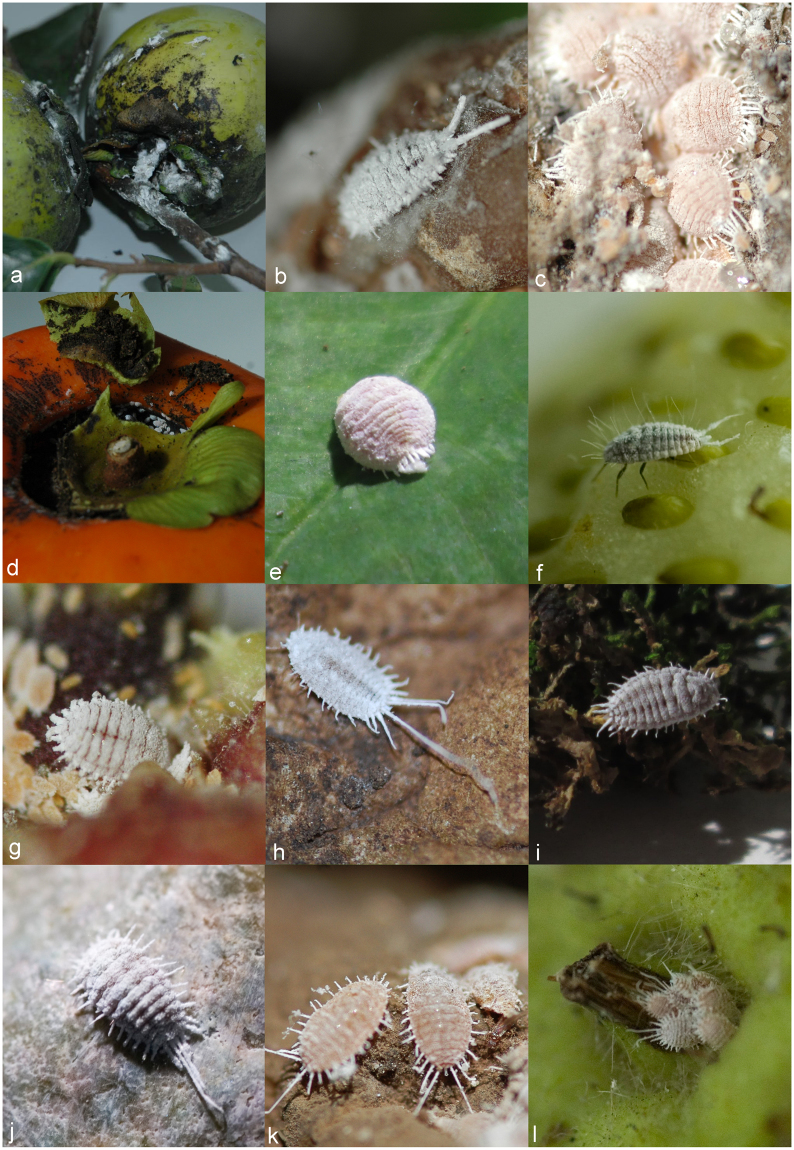
Mealybug species found in fruits on Serra Gaúcha region. (**a**,**b**) ♀ *Anisococcus granarae* (2.08–3.28 mm^55^); (**c**,**d**) ♀ *Dysmicoccus brevipes* (1.3–2.7 mm); (**e**) ♀ *Dysmicoccus sylvarum* (up to 4.4 mm^25^); (**f**) ♀ *Ferrisia meridionalis* (3.2–3.5 mm); (**g**) ♀ *Planococcus ficus* (1.4–3.2 mm^40^); (**h**) ♀ *Pseudococcus longispinus* (1.0–4.0 mm); (**i**,**j**) ♀ *Pseudococcus sociabilis* (1.9–3.2 mm^43^); (**k**,**l**) ♀ *Pseudococcus viburni* (1.8–3.5 mm^43^).

In total, 412 specimens were studied. Morphological examination of the voucher specimens led to identification of 22 mealybug species (Table 1). Photographs of live adult female specimens were obtained for the main mealybug taxa found in the Serra Gaúcha region (Fig. 1).

**Table 1:** Morphological identification of mealybugs (Hemiptera: Pseudococcidae) infesting fruit crops in southern Brazil.

Mealybug species	Host plant
*Anisococcus granarae* Pacheco da Silva & Kaydan	Persimmon
*Chorizococcus nakaharai* Williams & Granara de Willink	Persimmon
*Dysmicoccus brevipes* (Cockerell)	Grapes, persimmon, weeds (*Artemisia verlotorum* Lamotte, *Rumex* sp., *Trifolium* sp.)
*Dysmicoccus sylvarum* Williams & Granara de Willink	Grapes, persimmon, weeds (*Rumex* sp.)
*Dysmicoccus texensis* (Tinsley)	Persimmon
*Ferrisia kaki* Kaydan & Pacheco da Silva	Persimmon
*Ferrisia meridionalis* Williams	Persimmon, strawberry, weeds (*Conyza bonariensis* L.)
*Ferrisia terani* Williams & Granara de Willink	Persimmon
*Ferrisia williamsi* Kaydan & Gullan	Persimmon
*Nipaecoccus jacarandae* Williams & Granara de Willink	Persimmon
*Paracoccus galzerae* Pacheco da Silva & Kaydan	Weeds (Conyza bonariensis L.)
*Phenacoccus gregosus* Williams & Granara de Willink	Persimmon
*Phenacoccus* near *tucumanus*	Persimmon
*Planococcus citri* (Risso)	Persimmon
*Planococcus ficus* (Signoret)	Grapes
*Pseudococcus longispinus* (Targioni Tozzetti)	Grapes
*Pseudococcus meridionalis* Prado	Persimmon, weeds (*Conyza bonariensis* L.)
*Pseudococcus nakaharai* Gimpel & Miller	Persimmon
*Pseudococcus rosangelae* Pacheco da Silva & Kaydan	Persimmon
*Pseudococcus sociabilis* Hambleton	Persimmon
*Pseudococcus viburni* (Signoret)	Apple, grapes, persimmon, strawberry, weeds (*Artemisia verlotorum* Lamotte, *Rumex* sp.)
*Pseudococcus* near *maritimus*	Persimmon

Some specimens identified as *Pseudococcus* near *maritimus* were found to be close to *Pseudococcus maritimus* (Ehrhorn), differing mainly in the absence of one characteristic pair of oral ring tubular ducts next to the anterior ostioles. The concentrations of larger tubular ducts on the body margin and of multilocular disc pores on abdominal segment VII were quite variable in specimens identified as *D*. *sylvarum*, differing from the illustration in Williams and Granara de Willink (1992). Some individuals seemed to be close to *D*. *umbambae* Granara de Willink^24^, differing from the original description in (i) the absence of discoidal pores associated with the eyes and (ii) the absence of discoidal pores on the dorsal midline of all abdominal segments. The morphological analyses of *Phenacoccus* specimens was particularly complex and revealed the need for a much deeper analysis of this genus. Intraspecific differences were observed between our samples and the original descriptions of several taxa. Specimens identified as *Ph*. *gregosus* present some morphological divergences from the illustration in Williams & Granara de Willink (1992)^25^ in (i) the absence of clusters of multilocular disc pores on head, (ii) absence of translucent pores from the hind femur, and (iii) fewer conical setae in the cerarii (2 to 4 instead of 5 to 9). These specimens also seem to be close to *Phenacoccus baccharidis* Williams, but differ in (i) the type of oral collar tubular ducts present and (ii) the presence of translucent pores on the hind femur (absent in the specimens collected here). For specimens assigned to *Ph*. near *tucumanus*, morphological divergence was observed in the (i) absence of quinquelocular pores on venter in this specimens and (ii) the size of the oral collar tubular ducts, there being only one size observed in this specimen.

### DNA characterization

**Figure 2:**
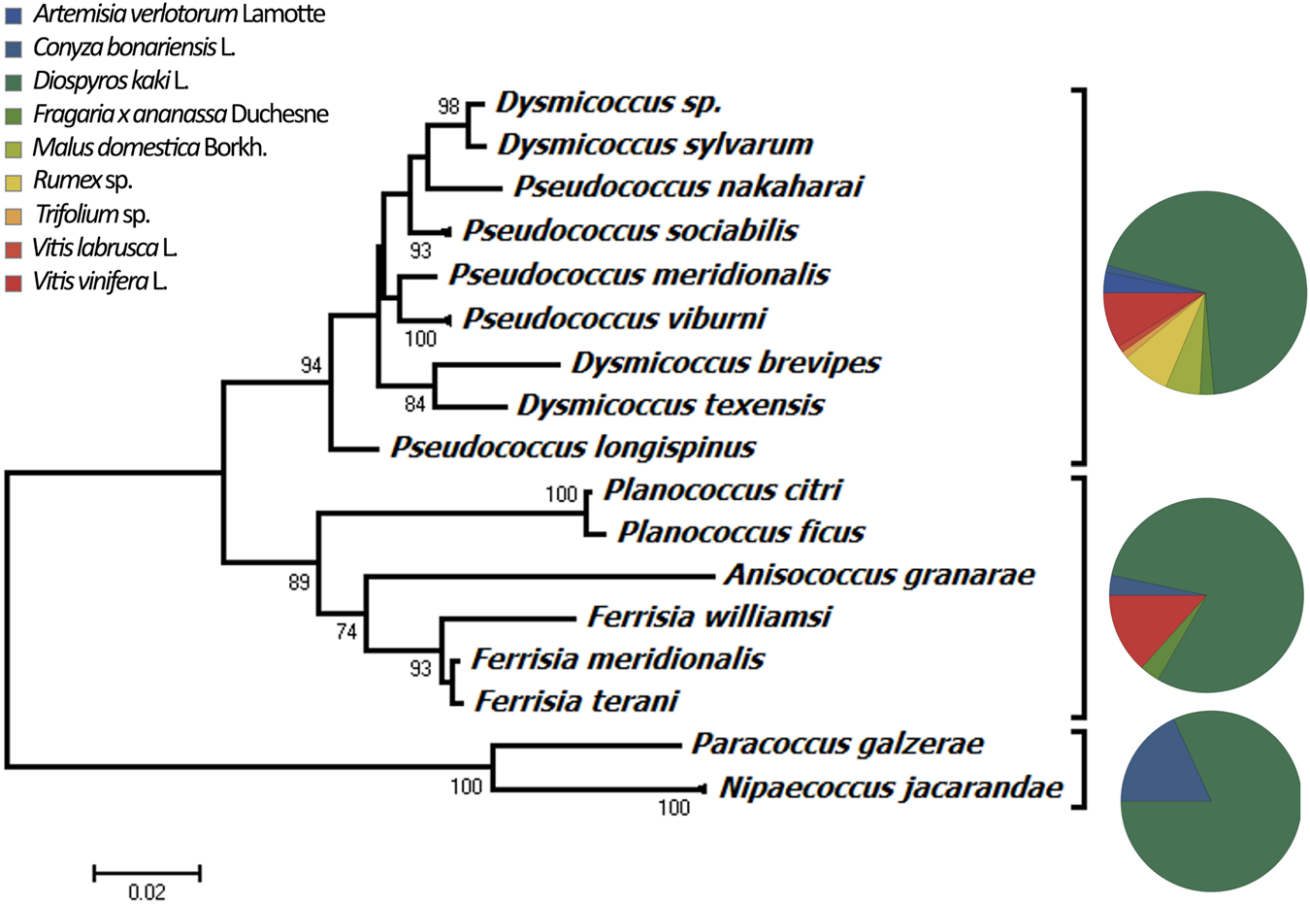
Neighbor-joining tree calculated using the Tajima-Nei distance (number of base substitutions per site). The rate of variation among sites was modeled with a gamma distribution (shape parameter = 0.36). All ambiguous positions were removed for each sequence pair and there were a total of 804 positions in the final dataset. Evolutionary analyses were conducted in MEGA7.

In total, 470 DNA sequences, including both COI and 28S gene regions, were obtained from 262 mealybug specimens. The primer pair for 28S provided 260 positive sequences, showing a much higher success rate (99.2%) than the COI gene region (79.4%). The 28S alignment was 804 bp long and resulted in 20 different haplotypes, whereas the COI alignment length was 680 bp long and resulted in 42 haplotypes. The sequence data allowed us to successfully distinguish 41 different multilocus haplotypes, forming 19 different taxonomic groups. The neighbor-joining tree generated using the Tajima-Nei distance based on the 28S sequences only resulted in 3 main clusters, and provides a visual representation of the host plant-mealybug associations present in the dataset (the NJ tree was not generated to provide phylogenetic information) (Fig. 2). Evolutionary analyses were conducted in MEGA7^26^.

DNA sequences could not be obtained from specimens identified based on morphology as *C*. *nakaharai*, *Ph*. *gregosus* and *Ps*. *rosangelae*. Nevertheless, DNA sequences were obtained from the specimens identified as *A*. *granarae*, *N*. *jacarandae*, *Pa*. *galzerae*, *Ph*. near *tucumanus*, *Ps*. *nakaharai* and *Ps*. *sociabilis* for the first time in this study (no previously recorded sequences from these species could be found in public databases). For the remaining species (*D*. *brevipes*, *D*. *sylvarum*, *D*. *texensis*, *F*. *meridionalis*, *F*. *terani*, *Pl*. *citri*, *Pl*. *ficus*, *Ps*. *longispinus*, *Ps*. *meridionalis* and *Ps*. *viburni*), the sequences obtained in this work were compared with those present in the NCBI GenBank database, and displayed a percentage similarity of between 99 and 100% with sequences assigned to the same species. The sequences obtained here for *Ps*. *meridionalis* are identical to those that were obtained in a previous study and assigned as *Pseudococcus* near *meridionalis* ^18^.

Populations of *Dysmicoccus* Ferris and *Pseudococcus* (Westwood) presented intraspecific variation. *Dysmicoccus brevipes* displaying multilocus haplotypes 28S04-COI05 were found on persimmon fruits and weeds located in two nearby cities, while *D*. *brevipes* displaying multilocus 28S04-COI06 were observed only on grape roots in Flores da Cunha. For *Dysmicoccus* sp., intraspecific variation was observed between mealybugs that developed on persimmon fruits in three different cities (Caxias do Sul, Farroupilha and Pinto Bandeira) (28S20-COI24) and mealybugs that were collected from weeds in Caxias do Sul (28S20-COI25). Four different multilocus haplotypes were obtained from different populations of *Ps*. *meridionalis*, two found on weeds and two found on persimmon. For *Ps*. *viburni* 11 multilocus haplotypes were observed. While individuals with 28S haplotype 28S06 displayed high variability at COI (10 COI haplotypes), individuals carrying the haplotype 28S08 displayed only one COI haplotype (COI33) even when collected from widely separated sites. Hence, out of the 11 multilocus haplotypes found in *Ps*. *viburni*, most genetic diversity was observed in the populations displaying 28S06. Interestingly, intraspecific genetic variation was observed in COI among mealybugs from the same site (on the same host plant and in the same city), e.g. for *A*. *granarae*, *D*. *sylvarum* and *Pa*. *galzerae*. Intraspecific variation was also observed in the 28S gene region for the species *N*. *jacarandae* (haplotypes 28S17 and 28S18) and *Ps*. *viburni* (haplotypes 28S06 and 28S08).

### Incidence of mealybug species among fruit crops

**Figure 3:**
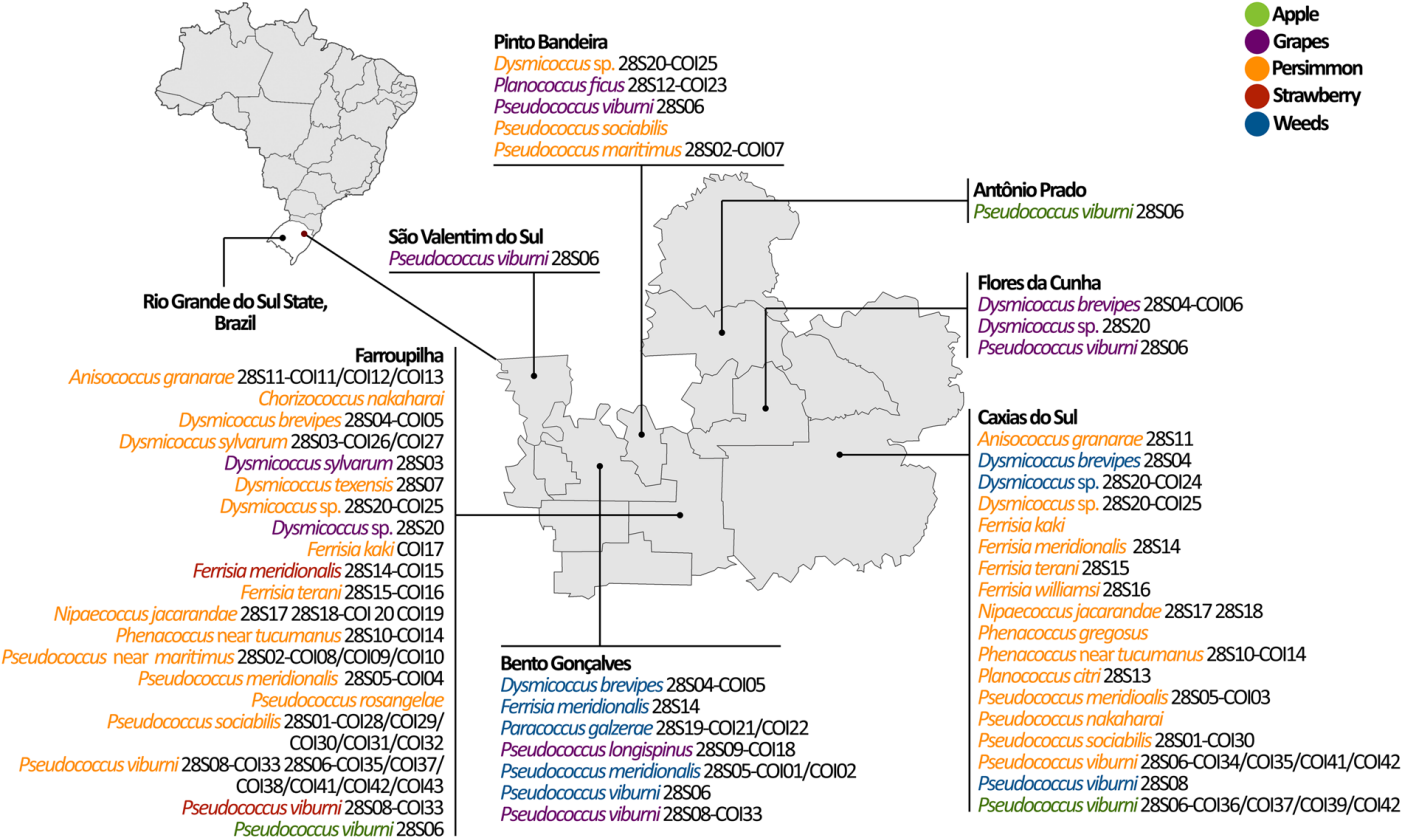
Summary of mealybug species distribution in the Serra Gaúcha region, RS, Brazil, and their multilocus haplotypes. Base map obtained from www.socioeconomicatlas.rs.gov.br; created in ArcGis 10.2.1 (www.arcgis.com) and modified in Photoshop CC 2014.

Mealybugs were found in about 50 production areas; four apple, 25 persimmon production areas and 17 vineyards (although in six of these mealybugs were found only on weeds) and only one strawberry field. The geographical distribution of mealybug species is highlighted in Fig. 3. Persimmon orchards revealed the highest incidence of mealybugs, with 56.8% (25 out of 44) of the surveyed orchards being infested. Twenty mealybug species were identified on persimmon trees (Table 1). Mealybugs occurred in 44.7% (17 out of 38) of grape sampling sites. Nine species were found in vineyards; among them, six were found on grapes and six were found on weeds (Table 1). Only two species namely, *F*. *meridionalis* and *Ps*. *viburni* were observed on strawberries, and only one production area (out of 8) was infested. Mealybugs were observed in only four (out of 34) of the apple orchards surveyed, and *Pseudococcus viburni* was the only species found.

## Discussion

The obscure mealybug, *Ps*. *viburni*, and the pineapple mealybug, *D*. *brevipes*, were the most common mealybug species found associated with fruit crops in the Serra Gaúcha region. *Pseudococcus viburni* was found on all the main fruits species surveyed, commonly in large numbers and widespread in the orchards and vineyards, resulting in aesthetic damage to the fruits. In contrast, *D*. *brevipes* was never found in high numbers; this species is an important pest in pineapple fields located in other regions of Brazil, specially due to transmission of pineapple mealybug wilt–associated virus^27^. In the Serra Gaúcha, *D*. *brevipes* was often found associated with grapes and weeds roots. *Pseudococcus viburni* is an important pest species infesting vineyards in southern part of South America, including Brazil and Chile^16,18,22^. The species is probably native to Brazil^28,29^, where it presents substantial morphological variation, in life and in slide-mounted specimens, and intraspecific genetic variation at both the 28S and COI genes^18,23^. In the Serra Gaúcha region, *Ps*. *viburni* was observed at a low infestation level in apple orchards. Nymphs and adults were found often in the calyx and sometimes also near the pedicel of the fruit. When these mealybugs were present, damage was caused by honeydew excretion resulting in the development of sooty molds. In other countries, including Argentina, Italy, New Zealand and South Africa, *Ps*. *viburni* is considered the most economically important species found infesting apple and pears^30–33^. This species was also the dominant species in strawberry fields (>than 99% of the specimens collected). To date, there have been only three records of mealybugs damaging strawberry plants, *Heliococcus bohemicus* (Šulc) and *Ps*. *viburni* in France^34^, and *Pseudococcus* sp. in Canada^35^. The damage can result in translucent spots on the leaves, which resembles a mosaic; malformation and dwarfing of the leaves; shortening of the petioles; and eventually plant death^35^.

In this study heavy infestations with the longtailed mealybug, *Ps*. *longispinus*, were observed in at least three seedling nurseries but never in commercial vineyards; whereas heavy infestations with the vine mealybug, *Planococcus ficus*, were observed in two vineyards. The vine and longtailed mealybugs are common species in Californian vineyards^10^. *Pseudococcus longispinus* is also found in vineyards in Chile^22^ and New Zealand^36^, while *Pl*. *ficus* is the main species on vines in other grape production regions^10,37,38^. However, neither of these species had been recorded causing damages in vineyards in Rio Grande do Sul before^16,18^. In previous studies, *Planococcus citri* was considered as the most economically important species in vineyards in southern Brazil^39^, however, molecular studies have not detected this species in vineyards in Rio Grande do Sul after 2010^18^, as confirmed in this work. Morphological identification of *Planococcus* species is extremely complex, mainly due to the low number of characters available for reliable identification and the high morphological variation that occurs in some species^40^. This has favored the use of molecular data to distinguish between similar taxa, such as *Pl*. *citri* and *Pl*. *ficus* ^41,42^. Unfortunately, the voucher specimens of previous studies were not saved for later analysis so the disappearance of *Pl*. *citri* from southern Brazilian vineyards remains unexplained.

Previous studies recorded mealybug in at least 50% of the persimmon orchards in the Serra Gaúcha region^15^, with more than one species found even within the same tree or fruit in the majority of the orchards. From the 20 species collected here on persimmon trees, three were found in the majority of orchards and can be considered as pests due to the high infestation rates and the damages caused to fruits: *A*. *granarae*, *Ps*. *sociabilis* and *Ps*. *viburni*. Adult females and nymphs of these species were found on the leaves and fruits (feeding directly on the fruits or in the fruit calyx). *Anisococcus granarae* was observed in several orchards in Farroupilha and Caxias do Sul, and high infestation rates were observed on the fruits at harvest time in at least two orchards. *Pseudococcus sociabilis* was the second most important species found in persimmon fruits in terms of abundance and damage. The honeydew excretion of this mealybug results in the development of sooty mold, which was observed coating the fruits at several localities.

The multilocus haplotypes and morphological identifications of the specimens collected in the present study were satisfactorily congruent except for *Dysmicoccus*. After initial morphological examination, the *Dysmicoccus* specimens were tentatively identified as *D*. *sylvarum*, despite some minor differences from the original description. Unexpectedly, molecular DNA analyses revealed the presence of two separate clades, suggesting the presence of cryptic species. Due to the limited number of adult females collected for the new clade, *Dysmicoccus* sp., it was not possible to achieve more conclusive results.

Here, several mealybug taxa are reported from Brazil for the first time. This is the first record of *C*. *nakaharai*, *F*. *williamsi*, *Ph*. *gregosus* and *Ps*. *nakaharai* in Brazilian territory; also the first record of *Ph*. *gregosus* and *Ps*. *nakaharai* in South America. *Pseudococcus nakaharai* has been recorded previously in Mexico, U.S.A., Guatemala and Japan, where it is often associated with at least 50 species of cacti^43^. *Ferrisia williamsi* was previously recorded on avocado *Persea americana* Mill. (Lauraceae) and ornamental trees in Colombia^44^, and *Phenacoccus gregosus* was found previously on four different host plant families (Amaranthaceae, Burseraceae, Euphorbiaceae and Fabaceae) in Mexico and Costa Rica^25^.

Our results revealed a high diversity of mealybug species in southern Brazil, particularly formed by species belonging to the “*Pseudococcus maritimus* complex” (see *Ps*. *meridionalis*, *Ps*. *nakaharai*, *Ps*. *rosangelae*, *Ps*. *sociabilis*, *Ps*. *viburni* and *Pseudococcus* near *maritimus*). The high level of genetic diversity further corroborates the previous hypothesis of a South American area of origin for *Ps*. *viburni* ^28,29^. New pests were identified for persimmon trees in the Serra Gaúcha region (*A*. *granarae*, *Ps*. *sociabilis* and *Ps*. *viburni*), and for grapevines (*Pl*. *ficus*) and strawberry fields (*Ps*. *viburni*). Serra Gaúcha fruit production is mainly based on small producers with yield destined for domestic trade; fruits destined for international trade are produced in other regions and states of Brazil. With the exception of exported grapes produced in the state of Pernambuco (where there is a low diversity of mealybugs^18,45^), little is known about mealybug infestation in the country. Further studies must be conducted in order to identify the mealybugs present in other fruit production areas of Brazil.

## Methods

### Mealybug sampling

Mealybugs were collected from commercial crops of apple (Rosaceae), grape (Vitaceae), persimmon (Ebenaceae) and strawberries (Rosaceae) chosen randomly in the Serra Gaúcha Region (Antônio Prado, Bento Gonçalves, Caxias do Sul, Farroupilha, Monte Belo and São Valentin do Sul) in RS, Brazil. The number of plants sampled was not standardized, but at least 30 plants per site were examined for mealybug infestations. A total of 78 mealybug populations were sampled; and considered as different populations when collected from different crop areas or different hosts within the same sampling area. Mealybug specimens for each population sample were collected from different parts of the plant (leaves, fruits, trunks and roots). For apples, grapes and persimmons, surveys were carried out between 2013 and 2015, preferably close to the harvesting time during November to April of each year, due to the higher mealybug abundance observed during this period. All developmental stages found (nymphs and/or females with/without ovisacs) were collected. In strawberry fields, sampling was carried out during September 2013 to July 2014 and September 2014 to May 2015. Adult females were stored in 95% ethanol at −20 °C. Nymphs were reared to adulthood for morphological species determination at the Entomology Laboratory of Embrapa Uva e Vinho, Bento Gonçalves, RS, Brazil. Pumpkins (*Curcubita maxima* Duchesne) and potato sprouts (*Solanum tuberosum* L.) were used as food substrates. Mealybugs were reared in plastic cages closed with voile tissue to allow ventilation. Paper towels were placed on the bottoms of the cages to prevent the accumulation of honeydew. Cages were kept at 25 ± 1 °C, relative humidity of 70 ± 10% and in a photophase of 14 h light and 10 h darkness.

### Morphological Identification

Slide mounting and identification of adult females were carried out at ANSES, *Laboratoire de la Santé des Végétaux*, Montferrier-sur-Lez, France and Plant Protection Department of Çukurova University, Adana, Turkey. The specimens were slide-mounted using Kosztarab and Kozár’s (1988)^46^ method with some modification (using distilled water after KOH and cleaning the specimens using a fine brush). Identification was done using an optical microscope (LEICA DM 2500 phase contrast compound microscope) and the keys of von Ellenrieder & Watson (2016)^47^; Kaydan & Gullan (2012)^44^; Granara de Willink (2009)^24^, Granara de Willink & Szumik (2007)^48^; Williams (2004)^49^; Gimpel & Miller (1996)^43^; Williams & Granara de Willink, (1992)^25^; Cox & Ben-Dov (1986)^50^ and McKenzie (1967)^51^. The slide-mounted specimens were stored in the Coccoidea Collection of the Museum Ramiro Gomes Costa Porto Alegre, Brazil (MRGC); Çukurova University Coccomorpha collection, Adana, Turkey (KPTC); Anses, *Laboratoire de la Santé des Végétaux*, Montferrier-sur-Lez, France (ANSES/LSV) and in the Entomological Collection of Embrapa Uva e Vinho, Bento Gonçalves, Brazil (CEEUV) (listed in the Supplementary Material, Table 1).

### Molecular characterization

The molecular characterization of mealybugs was performed at Sophia Agrobiotech Institut – INRA (*Institut National de Recherche Agronomique*), Sophia Antipolis, France. When possible, we analyzed at least three individuals from each population collected. DNA was extracted using DNeasy Blood and Tissue Kit (QIAGEN, Valencia, CA), based on the non-destructive methodology described by Malausa *et al*.^23^. Vouchers were kept in ethyl alcohol 70% for morphological analysis. DNA was amplified from two different loci: the HCO-LCO region of the cytochrome oxidase subunit 1 (mtDNA) and the 28S ribosomal gene (nuclear genome). Polymerase chain reactions were carried out using the Qiagen Multiplex PCR kit (QIAGEN, Valencia, CA), composed by 23 µL of reaction mix (1X Qiagen buffer, primers at 0.2 µM) and 2 µL of diluted DNA (1–20 ng of DNA). The primers (Forward, Reverse) C-28SLong-F 5′GAGAGTTMAASAGTACGTGAAAC3′ and C-28SLong-R 5′TCGGARGGAACCAGCTACTA3′ (28S-D2) were used for amplifying the 28S gene region^52^ and the primers PCO-F1 5′CCTTCAACTAATCATAAAAATATYAG3′ and Lep-R1 5′TAAACTTCTGGATGTCCAAAAAATCA3′ were used to characterize the COI gene region^53^. PCR reactions were performed as follows: initial denaturation for 15 minutes at 95 °C, 35 cycles of denaturation – hybridization – elongation (30 seconds at 95 °C for denaturation; 90 seconds at 58 °C for 28S and 54 °C for COI for hybridization; 60S at 72 °C for elongation), and a final extension for 10 minutes at 72 °C. PCR products were sent to Beckman Coulter Genomics (Takeley, United Kingdom) for bidirectional sequencing. Consensus sequences were constructed and checked in Seqscape v.27 (Applied Biosystems, Foster City, CA, USA). Alignments were edited manually in Bioedit v.7.02 (Hall, 1999)^54^. Sequences were compared between them by direct alignment, and with sequences previously described, using the search option MEGABLAST provided by the GenBank database (http://www.ncbi.nlm.gov/BLAST). New sequences were deposited in GenBank under the accession numbers: KY565026 to KY565046 for 28S and KY687861 to KY687903 for COI. Before carrying out the phylogenetic analyses from the 28S gene region only, all ambiguous positions were removed using GBLOCKS with default parameters, leaving a total of 804 positions in the final dataset. Model selection of nucleotide substitution was performed with MEGA7 Kumar *et al*.^26^ according to BIC scores (Bayesian Information Criterion) and AICc value (Akaike Information Criterion, corrected). The Tajima-Nei model, plus rate variation among sites modeled with a gamma distribution (shape parameter = 0.36), was selected for the 28S alignment. A Neighbor-joining (NJ) phylogenetic tree was built using MEGA7. Bootstrap branch support values were calculated with 500 replicates.

## Electronic supplementary material

Supplementary information
