# Supplementary material for: Integrative taxonomy methods reveal high mealybug (Hemiptera: Pseudococcidae) diversity in southern Brazilian fruit crops
Source: Sci Rep. 2017 Nov 16;7:15741. doi: 10.1038/s41598-017-15983-5 (PMC5691041; doi:10.1038/s41598-017-15983-5)
Supplement: Supplementary file 1 — Supplementary information [file 41598_2017_15983_MOESM1_ESM.doc]

**Integrative taxonomy methods reveal high mealybug (Hemiptera: Pseudococcidae) diversity in southern Brazilian fruit crops**

Vitor C. Pacheco da Silva; Mehmet Bora Kaydan; Thibaut Malausa; Jean-François Germain; Ferran Palero; Marcos Botton

**Supplementary material**

| **Table 1: List of populations sampled, geographic origin, host plant, identification and slide number.** | | | | |
| --- | --- | --- | --- | --- |
| Population | City | Host Plant | Slide number | Identity (morphological + molecular) |
|  |  |  |  |  |
| M01 | Farroupilha | *Diospyros kaki* L. | A1401229 | *Ferrisia terani* Williams & Granara de Willink |
| M02 | Bento Gonçalves | *Vitis vinifera* L. |  | *Pseudococcus viburni* (Signoret) |
| M03 | Farroupilha | *Diospyros kaki* L. | A1401251, VCP405, VCP406 | *Pseudococcus viburni* (Signoret) |
| M04 | Caxias do Sul | *Malus domestica* Borkh. | A1401250, VCP416, VCP417 | *Pseudococcus viburni* (Signoret) |
| M05 | Farroupilha | *Diospyros kaki* L. | VCP407 | *Pseudococcus viburni* (Signoret) |
| M06 | Farroupilha | *Diospyros kaki* L. | A1401216, A1401215, A1401217, A1401239, A1401240, A1401241, VCP414, VCP415, VCP404 | *Pseudococcus sociabilis* Hambleton |
| M07 | Farroupilha | *Malus domestica* Borkh. |  | *Pseudococcus viburni* (Signoret) |
| M08 | Farroupilha | *Diospyros kaki* L. | A1401225 | *Pseudococcus meridionalis* Prado |
|  |  |  | A1401226 | *Nipaecoccus jacarandae* Williams & Granara de Willink |
| M10 | Farroupilha | *Diospyros kaki* L. | A140232, A1401233 | *Dysmicoccus sylvarum* Williams & Granara de Willink |
| M15 | Farroupilha | *Diospyros kaki* L*.* | A1401218, A1401219, A1401220 | *Anisococcus granarae* Pacheco da Silva & Kaydan |
| M16 | Farroupilha | *Diospyros kaki* L. | A1401221 | *Pseudococcus sociabilis* Hambleton |
| M18 | Pinto Bandeira | *Diospyros kaki* L. | A1401227 | *Dysmicoccus* sp. |
| M19 | Farroupilha | *Diospyros kaki* L. | A1401234, A1401235 | *Dysmicoccus brevipes* (Cockerell) |
| M20 | Pinto Bandeira | *Diospyros kaki* L. | VCP449 | *Pseudococcus sociabilis* Hambleton |
| M21 | Antônio Prado | *Malus domestica* Borkh. |  | *Pseudococcus viburni* (Signoret) |
| M22 | Bento Gonçalves | *Conyza bonariensis* L. | A1401237, A1401238, A1401242, MRGC2269, MRGC2270 | *Paracoccus galzerae* Pacheco da Silva & Kaydan |
| M23 | Caxias do Sul | *Rumex* sp. and *Artemisia verlotorum*  Lamotte |  | *Pseudococcus viburni* (Signoret) |
|  |  |  |  | *Dysmicoccus brevipes* (Cockerell) |
| M24 | Caxias do Sul | *Rumex* sp. and *Artemisia verlotorum*  Lamotte | VCP444, VCP445 | *Pseudococcus viburni* (Signoret) |
| M25 | Farroupilha | *Diospyros kaki* L. | VCP448 | *Ferrisia terani* Williams & Granara de Willink |
| M26 | Pinto Bandeira | *Vitis vinifera* L. | A1401243 | *Planococcus ficus* (Signoret) |
| M29 | Pinto Bandeira | *Vitis vinifera* L. | A1401245, A1401248 | *Planococcus ficus* (Signoret) |
|  |  |  | VCP328 | *Pseudococcus* near *maritimus* |
| M31 | Farroupilha | *Fragaria x ananassa*  Duchesne | A1401254, A1401252 | *Pseudococcus viburni* (Signoret) |
| F | Farroupilha | *Fragaria x ananassa* Duchesne | A1401230, A1401231 | *Ferrisia meridionalis* Williams |
| C12 | Bento Gonçalves | *Vitis vinifera* L. |  | *Pseudococcus viburni* (Signoret) |
| CIV | Caxias do Sul | *Malus domestica* Borkh. |  | *Pseudococcus viburni* (Signoret) |
| M35 | Antônio Prado | *Malus domestica* Borkh. | VCP008 | *Pseudococcus viburni* (Signoret) |
| M36 | Flores da Cunha | *Vitis vinifera*  L. | VCP009 | *Dysmicoccus brevipes* (Cockerell) |
|  |  |  | VCP010 | *Pseudococcus viburni* (Signoret) |
| M38 | Bento Gonçalves | *Vitis vinifera*  L. | VCP012, VCP013 | *Planococcus ficus* (Signoret) |
| M40 | São Valentin do Sul | *Vitis labrusca*  L. | VCP014, VCP015 | *Pseudococcus viburni* (Signoret) |
| M41 | Farroupilha | *Fragaria x ananassa*  Duchesne | VCP016, VCP017 | *Pseudococcus viburni* (Signoret) |
| M43 | Bento Gonçalves | *Rumex* sp. | VCP021 | *Pseudococcus viburni* (Signoret) |
| M44 | Bento Gonçalves | *Conyza bonariensis*  L. | VCP022, VCP023, VCP024 | *Ferrisia meridionalis* Prado |
| M45 | Bento Gonçalves | *Trifolium* sp. | VCP025, VCP026, VCP027 | *Dysmicoccus brevipes* (Cockerell) |
| M46 | Bento Gonçalves | *Conyza bonariensis*  L. | VCP028, VCP031, VCP029, VCP311, VCP312 | *Pseudococcus meridionalis* Prado |
| M47 | Bento Gonçalves | *Conyza bonariensis*  L. | VCP030, VCP308, VCP309, VCP310, VCP032, VCPC033, VCP034, VCP313, VCP314, VCP315 | *Paracoccus galzerae* Pacheco da Silva & Kaydan |
| M53 | Pinto Bandeira | *Vitis vinifera*  L. | VCP035 | *Planococcus ficus* (Signoret) |
| M54 | Flores da Cunha | *Vitis vinifera*  L. | VCP036, VCP037, VCP038, VCP408, VCP409 | *Dysmicoccus sylvarum* Williams & Granara de Willink |
| M57 | Farroupilha | *Diospyros kaki*  L. | VCP039 | *Pseudococcus sociabilis* Hambleton |
| M58 | Farroupilha | *Diospyros kaki* L. | VCP041 | *Dysmicoccus sylvarum* Williams & Granara de Willink |
| M59 | Bento Gonçalves | *Vitis vinifera*  L. | VCP042, VCP043, VCP044 | *Pseudococcus viburni* (Signoret) |
| M60 | Farroupilha | *Diospyros kaki* L. | VCP046, VCP316 | *Dysmicoccus* sp. |
|  |  |  | VCP047 | *Dysmicoccus sylvarum* Williams & Granara de Willink |
| M61 | Bento Gonçalves | *Vitis vinifera*  L. | VCP048 | *Pseudococcus longispinus* (Targioni Tozzetti) |
| M62 | Farroupilha | *Diospyros kaki*  L. | VCP051 | *Dysmicoccus* sp. |
|  |  |  | VCP050, VCP052 | *Dysmicoccus sylvarum* Williams & Granara de Willink |
| M63 | Farroupilha | *Diospyros kaki*  L. | VCP053, VCP054, VCP423, VCP424, MRGC2263, MRGC2264, MRGC2265 | *Anisococcus granarae* Pacheco da Silva & Kaydan |
| M66 | Farroupilha | *Diospyros kaki*  L. | VCP060, VCP061 | *Dysmicoccus brevipes* (Cockerell) |
| M68 | Farroupilha | *Diospyros kaki* L. | VCP062 | *Dysmicoccus texensis* (Tinsley) |
|  |  |  | VCP063 | *Dysmicoccus* sp. |
| M69 | Farroupilha | *Diospyros kaki*  L. | VCP064 | *Dysmicoccus brevipes* (Cockerell) |
| M70 | Farroupilha | *Diospyros kaki* L. | VCP065, VCP066 | *Anisococcus granarae* Pacheco da Silva & Kaydan |
|  |  |  | VCP322 | *Nipaecoccus jacarandae* Williams & Granara de Willink |
|  |  |  | VCP072  VCP321 | *Pseudococcus meridionalis* Prado  *Pseudococcus sociabilis* Hambleton |
|  |  |  | VCP067 | *Pseudococcus viburni* (Signoret) |
|  |  |  | VCP069 | *Pseudococcus* near *maritimus* |
|  |  |  | VCP070 |  |
| M76 | Farroupilha | *Diospyros kaki*  L. | VCP073 | *Pseudococcus viburni* (Signoret) |
|  |  |  | VCP325, VCP326, VCP327 | *Pseudococcus* near *maritimus* |
| M77 | Caxias do Sul | *Diospyros kaki*  L. | VCP076 | *Dysmicoccus sylvarum* Williams & Granara de Willink |
| M78 | Caxias do Sul | *Diospyros kaki*  L. | VCP329 | *Nipaecoccus jacarandae* Williams & Granara de Willink |
|  |  |  | VCP078, VCP330 | *Pseudococcus meridionalis* Prado |
|  |  |  | VCP080 | *Pseudococcus viburni* (Signoret) |
| M79 | Farroupilha | *Diospyros kaki*  L. | VCP422 | *Dysmicoccus* sp. |
|  |  |  | VCP340 | *Ferrisia kaki* Kaydan & Pacheco da Silva |
|  |  |  | VCP333, VCP081 | *Nipaecoccus jacarandae* Williams & Granara de Willink |
|  |  |  | VCP102 | *Phenacoccus* near *tucumanus* Granara de Willink |
|  |  |  | VCP083 | *Pseudococcus sociabilis* Hambleton |
|  |  |  | VCP341, VCP342, VCP343, VCP095, VCP103 | *Pseudococcus viburni* (Signoret) |
|  |  |  | VCP082, VCP331 | *Pseudococcus* near *maritimus* |
| M80 | Farroupilha | *Diospyros kaki*  L. | VCP084, VCP085, VCP089, VCP337, VCP338, VCP428, VCP329, VCP432 | *Anisococcus granarae* Pacheco da Silva & Kaydan |
|  |  |  | MRGC2262 | *Pseudococcus rosangelae* Pacheco da Silva & Kaydan |
|  |  |  | VCP335, VCP336, VCP427, VCP430 | *Pseudococcus sociabilis* Hambleton |
|  |  |  | VCP094, VCP334, VCP339, VCP431, VCP433 | *Pseudococcus viburni* (Signoret) |
| M81 | Caxias do Sul | *Diospyros kaki*  L. | VCP096 | *Nipaecoccus jacarandae* Williams & Granara de Willink |
|  |  |  | VCP093 | *Pseudococcus viburni* (Signoret) |
| M84 | Farroupilha | *Diospyros kaki*  L. | VCP107 | *Ferrisia terani* Williams & Granara de Willink |
|  |  |  | VCP345 | *Pseudococcus viburni* (Signoret) |
|  |  |  | VCP344 | *Pseudococcus* near *maritimus* |
| M85 | Caxias do Sul | *Diospyros kaki* L. | VCP108 | *Ferrisia meridionalis* Williams |
|  |  |  | VCP110 | *Ferrisia williamsi* Kaydan & Gullan |
|  |  |  | VCO120 | *Phenacoccus gregosus* Williams & Granara de Willink |
|  |  |  | VCP121 | *Pseudococcus meridionalis* Prado |
|  |  |  | VCP346, VCP425 | *Pseudococcus viburni* (Signoret) |
| M86 | Farroupilha | *Diospyros kaki*  L. | VCP113, VCP114, VCP119, VCP351, VCP434, VCP437, VCP112, VCP142 | *Anisococcus granarae* Pacheco da Silva & Kaydan |
|  |  |  | VCP111, VCP352, VCP355, VCP356, VCP357, VCP435, VCP436 | *Ferrisia kaki* Kaydan & Pacheco da Silva |
|  |  |  | VCP116, VCP141 | *Ferrisia terani* Williams & Granara de Willink |
|  |  |  | VCP115, VCP118 | *Nipaecoccus jacarandae* Williams & Granara de Willink |
|  |  |  | VCP350 | *Pseudococcus sociabilis* Hambleton |
| M88 | Caxias do Sul | *Diospyros kaki*  L. | VCP129, VCP131 | *Anisococcus granarae* Pacheco da Silva & Kaydan |
|  |  |  | VCP125, MRGC2266, MRGC2267 | *Ferrisia kaki* Kaydan & Pacheco da Silva |
|  |  |  | VCP126 | *Ferrisia williamsi* Kaydan & Gullan |
|  |  |  | VCP132, VCP133, VCP134, VCP124 | *Nipaecoccus jacarandae* Williams & Granara de Willink |
|  |  |  | VCP128 | *Phenacoccus* near *tucumanus* Granara de Willink |
|  |  |  | VCP438, VCP122, VCP123, VCP127, VCP130 | *Pseudococcus viburni* (Signoret) |
| M89 | Caxias do Sul | *Diospyros kaki* L. | VCP135 | *Ferrisia kaki* Kaydan & Pacheco da Silva |
| M90 | Caxias do Sul | *Diospyros kaki* L. | VCP137 | *Ferrisia terani* Williams & Granara de Willink |
|  |  |  | VCP138 | *Pseudococcus viburni* (Signoret) |
| M91 | Caxias do Sul | *Diospyros kaki* L. | VCP091 | *Pseudococcus meridionalis* Prado |
| M93 | Farroupilha | *Diospyros kaki* L. | VCP147 | *Nipaecoccus jacarandae* Williams & Granara de Willink |
|  |  |  | VCP148  MBK | *Pseudococcus* near *maritimus*  *Chorizococcus nakaharai* Williams & Granara de Willink |
| M94 | Farroupilha | *Diospyros kaki* L. | VCP150, VCP152 | *Pseudococcus sociabilis* Hambleton |
| M95 | Farroupilha | *Diospyros kaki* L. | VCP157 | *Ferrisia kaki* Kaydan & Pacheco da Silva |
|  |  |  | VCP359 | *Nipaecoccus jacarandae* Williams & Granara de Willink |
|  |  |  | VCP358 | *Pseudococcus viburni* (Signoret) |
| M96 | Pinto Bandeira | *Diospyros kaki* L. | VCP092 | *Pseudococcus viburni* (Signoret) |
| M97 | Farroupilha | *Diospyros kaki* L. | VCP360, VCP402, VCP160, VCP158, VCP159 | *Dysmicoccus sylvarum* Williams & Granara de Willink |
|  |  |  | VCP403 | *Pseudococcus sociabilis* Hambleton |
| M98 | Farroupilha | *Diospyros kaki* L. | VCP162 | *Dysmicoccus brevipes* (Cockerell) |
| M99 | Farroupilha | *Diospyros kaki* L*.* | VCP163, VCP164 | *Pseudococcus sociabilis* Hambleton |
| M100 | Caxias do Sul | *Diospyros kaki* L. | VCP166 | *Anisococcus granarae* Pacheco da Silva & Kaydan |
|  |  |  | VCP171, VCP174 | *Ferrisia kaki* Kaydan & Pacheco da Silva |
|  |  |  | VCP167, VCP364, VCP365 | *Phenacoccus* near *tucumanus* Granara de Willink |
|  |  |  | VCP173 | *Pseudococcus nakaharai* Gimpel & Miller |
|  |  |  | VCP169, VCP363 | *Pseudococcus sociabilis* Hambleton |
|  |  |  | VCP168, VCP170, VCP175, VCP361, VCP362 | *Pseudococcus viburni* (Signoret) |
| M101 | Farroupilha | *Diospyros kaki* L. | VCP191, VCP192, VCP367, VCP368, VCP369, VCP370, VCP371, VCP373, VCP375, VCP376, VCP377, VCP382, VCP383, VCP385, VCP386, VCP388, VCP389, VCP390, VCP391, VCP392. VCP393, VCP395, VCP190 | *Anisococcus granarae* Pacheco da Silva & Kaydan |
|  |  |  | VCP193 | *Phenacoccus* near *tucumanus* Granara de Willink |
|  |  |  | VCP182, VCP194, VCP195, VCP177, VCP178, VCP179, VCP189, VCP366, VCP374, VCP381, VCP384, VCP387, VCP394 | *Pseudococcus sociabilis* Hambleton |
|  |  |  | VCP181, VCP188, VCP372, VCP378, VCP379 | *Pseudococcus viburni* (Signoret) |
| M102 | Farroupilha | *Diospyros kaki* L. | VCP397 | *Anisococcus granarae* Pacheco da Silva & Kaydan |
|  |  |  | VCP185, VCP398 | *Pseudococcus sociabilis* Hambleton |
|  |  |  | VCP184, VCP186 | *Pseudococcus viburni* (Signoret) |
| M104 | Farroupilha | *Diospyros kaki* L. | VCP139 | *Pseudococcus sociabilis* (Hambleton) |
| M105 | Caxias do Sul | *Diospyros kaki* L*.* | VCP196 | *Dysmicoccus sylvarum* Williams & Granara de Willink |
|  |  |  | VCP197 | *Planococcus citri* (Risso) |
| M108 | Caxias do Sul | *Rumex* sp. | VCP200, VCP411, VCP412 | *Dysmicoccus* sp. |
|  |  |  | VCP199 | *Dysmicoccus sylvarum* Williams & Granara de Willink |
| M110 | Bento Gonçalves | *Rumex* sp. | VCP203, VCP204, VCP205 | *Dysmicoccus brevipes* (Cockerell) |
| M111 | Farroupilha | *Diospyros kaki*  L. | VCP207 | *Dysmicoccus brevipes* (Cockerell) |
| *A = ANSES collection, *MRGC = Museum Ramiro Gomes Costa *VCP = Entomological Collection of Embrapa Grape and Wine. | | | | |
